# Optimized Strategies for Managing Abdominal Hydatid Cysts and Their Complications

**DOI:** 10.3390/diagnostics14131346

**Published:** 2024-06-25

**Authors:** Alin Mihetiu, Dan Bratu, Dan Sabau, Octavian Nastase, Alexandra Sandu, Ciprian Tanasescu, Adrian Boicean, Cristian Ichim, Samuel Bogdan Todor, Dragos Serban, Adrian Hasegan

**Affiliations:** 1 Second Surgical Department, Lucian Blaga University of Sibiu, County Clinical Emergency Hospital of Sibiu, 550024 Sibiu, Romania; alin_mihetiu@yahoo.com (A.M.); alexandrasandu96@yahoo.ro (A.S.); 2 Faculty of Medicine, Lucian Blaga University of Sibiu, 550169 Sibiu, Romaniaadrian.boicean@ulbsibiu.ro (A.B.); cristian.ichim@ulbsibiu.ro (C.I.);; 3 Radiology Department, County Clinical Emergency Hospital of Sibiu, 550245 Sibiu, Romania; 4 First Surgical Department, Lucian Blaga University of Sibiu, County Clinical Emergency Hospital of Sibiu, 550024 Sibiu, Romania; ciprian.tanasescu@ulbsibiu.ro; 5 Faculty of Medicine, Carol Davila University of Medicine and Pharmacy, Bucharest Emergency University Hospital, 050474 Bucharest, Romania; dragos.serban@umfcd.ro; 6 Urology Department, Lucian Blaga University of Sibiu, County Clinical Emergency Hospital of Sibiu, 550024 Sibiu, Romania; adrian.hasegan@ulbsibiu.ro

**Keywords:** hepatic hydatid cyst, laparoscopic surgery, open surgery, ERCP, PAIR

## Abstract

Hepatic hydatid cysts are an example of a zoonosis with global distribution, but with endemic characteristics in certain geographic areas. Known since ancient times, this parasitic infection predominantly affecting the liver and lungs remains a challenge today in terms of diagnosis and the pharmacological, radiological, endoscopic, or surgical therapy. This study analyzed the complications associated with different procedures for treating hydatid cysts in 76 patients admitted to the County Clinical Emergency Hospital of Sibiu. Complications occurred in 18 patients (23.7%), with no significant correlation to age, gender, or residency (urban or rural). Patients undergoing open surgery exhibited the highest complication rate (61.1%) compared to those treated with other procedures. The most frequent complication was biliary duct rupture, occurring in 22.7% of cases. Our findings indicate that the presence of complications significantly prolongs hospitalization time [t df (75) = 12.14, *p* < 0.001]. Based on these findings, we conclude that the surgical approach for hydatid cysts should be meticulously tailored to each patient’s specific circumstances to reduce the risk of complications and improve clinical outcomes.

## 1. Introduction

The hydatid cyst is a zoonosis caused by infection with the *Echinococcus* tapeworm, most commonly the granulosus and multilocularis variants. According to the World Health Organization (WHO), it is an infection that affects about 1 million people worldwide and leads to a substantial financial toll of billions of US dollars in annual expenses [1].

The condition is more commonly endemic, with distribution primarily in certain geographic areas, where animal husbandry is widespread, such as the Mediterranean region, the Balkans, North Africa, the Middle East, Asia, South America, Australia and New Zealand. The disease has been known since ancient times, with descriptions of it compiled in the *Corpus Hippocraticum* and works by Celsus and Galen [2,3,4,5,6,7].

Hydatid disease is a prevalent zoonotic infection, most commonly found in regions where traditional animal are widespread. The adult *Echinococcus granulosus*, measuring between 2 and 7 mm in length, and the *Echinococcus multilocularis* (1.2–4.5 mm long) reside within the small intestine of its definitive host. Gravid proglottids release eggs that are excreted in the host’s feces, rendering them immediately infectious. Upon ingestion by an appropriate intermediate host, these eggs hatch in the small intestine, releasing six-hooked oncospheres that penetrate the intestinal wall and disseminate through the circulatory system to various organs, predominantly the liver and lungs. In these locations, the oncospheres mature into thick-walled hydatid cysts, which progressively enlarge and generate protoscolices and daughter cysts within their interior. The definitive host becomes infected by ingesting the organs of the intermediate host that contain these cysts. Once ingested, the protoscolices evaginate, adhere to the intestinal mucosa and develop into adult stages within a period of 32 to 80 days. Humans act as aberrant intermediate hosts and become infected through the ingestion of eggs. These eggs release oncospheres in the human intestine, leading to the formation of hydatid cysts in various organs. In the event of cyst rupture, the liberated protoscolices may lead to the formation of secondary cysts in other anatomical sites, a condition known as secondary echinococcosis [8,9,10].

In the early 1990s, the treatment of hydatid cysts entered a new stage of minimally invasive procedures, through laparoscopic surgery and radiologically guided PAIR (puncture, aspiration, injection, reaspiration). Results have significantly improved compared to open surgery, allowing for the approach of multiple localizations, difficult localizations, or extrahepatic ones. Handling of complications of hydatid disease often remains within the realm of open surgery [11,12].

PAIR, credited as a minimally invasive modality with wide applicability, is however limited by the risk of spillage, indication for a single-cyst approach, cyst contents and increased recurrence rate. Laparoscopic surgery, more aggressive than PAIR, has its limitations in disseminated abdominal hydatidosis and presents higher postoperative complications and hospitalization compared to interventional radiological approaches. Robotic surgery for hydatid cysts, although in its early stages, offers prospects of results at least similar to laparoscopy. Recently, radiofrequency or microwave ablation techniques have shown promise in non-interventional resolution of this pathology. ERCP (Endoscopic Retrograde Cholangiopancreatography) achieves curative results in cases with compressive, obstructive, inflammatory complications, or in ruptures of hydatid cysts into the bile ducts, also having an adjuvant effect in postoperative complications like bile leak [13,14,15]. However, the medical team must consider the risk of severe complications, such as post-ERCP pancreatitis [16].

Approximately 40% of hydatid cysts will develop complications during their evolution. This percentage is significant, and early diagnosis and treatment of this condition are preferable to presenting it in a complicated phase of hydatid disease. The liver is the most affected organ by *Echinococcus granulosus*, representing approximately 70% of localizations in humans, with the right lobe being involved in 85% of cases. This distribution results in frequent liver-related complications, manifesting as hepatic parenchymal involvement, vascular and biliary structures of the liver, and less commonly, neighboring organs. The most frequent complications include cyst superinfection, rupture into the biliary tract, mass effect, peritoneal dissemination and recurrence [17,18,19].

The primary objective of this research is to provide a detailed comparison of the complication rates associated with various procedures for treating hydatid cysts, with a focus on identifying which methods are associated with higher risks. By analyzing data from 76 patients treated at the County Clinical Emergency Hospital of Sibiu, this study seeks to identify patterns in complication incidence and their correlation with the treatment type.

## 2. Material and Methods

We conducted a retrospective analysis between January 2017 and January 2024, on the patients admitted to the Surgical Departments of the Emergency County Hospital of Sibiu with abdominal hydatid cysts. The inclusions criteria were

- Patients aged over 18 years, with hydatid cysts, regardless of abdominal localization or complication status, who underwent surgical treatment during the admission.

The exclusion criteria were

- Pediatric patients, patients who were admitted but did not undergo any therapeutic procedures;
- Patients who died before receiving any surgical treatment.

We documented demographic data of the patients included in the study, the cyst location, the existence of any complication at admission, the type of surgical procedure and postoperative outcomes.

### 2.1. Operative and Postoperative Data

The therapeutic management approaches identified were open surgery via laparotomy, laparoscopic approach with standard instruments, laparoscopic approach with dedicated instruments and ERCP.

Preoperative preparation was carried out with Albendazole 400 mg twice a day a month prior to surgery in all cases scheduled by appointment. Patients who presented to the surgical service as emergencies did not receive prophylactic antiparasitic treatment, which was initiated postoperatively. Among the group with complications of hydatid disease, 42.86% of patients received antiparasitic treatment, being admitted by appointment for cases of hydatid recurrence and one case of compression on the right branch of the portal vein. The duration from admission to surgical intervention was 1.89 ± 0.87 days, 1.44 ± 0.86 days for cases with complications of the hydatid cyst and 1.64 ± 1 days for cases without complications. It is observed that the average duration until surgical intervention was shorter for cases with complications compared to those without complications, but comparative analysis of these parameters did not show statistical significance (*p* > 0.05). The surgical approaches used were open surgery, standard laparoscopic approach, and laparoscopic approach with specially adapted instruments for hydatid disease.

#### 2.1.1. Open Surgery

**Figure 1:**
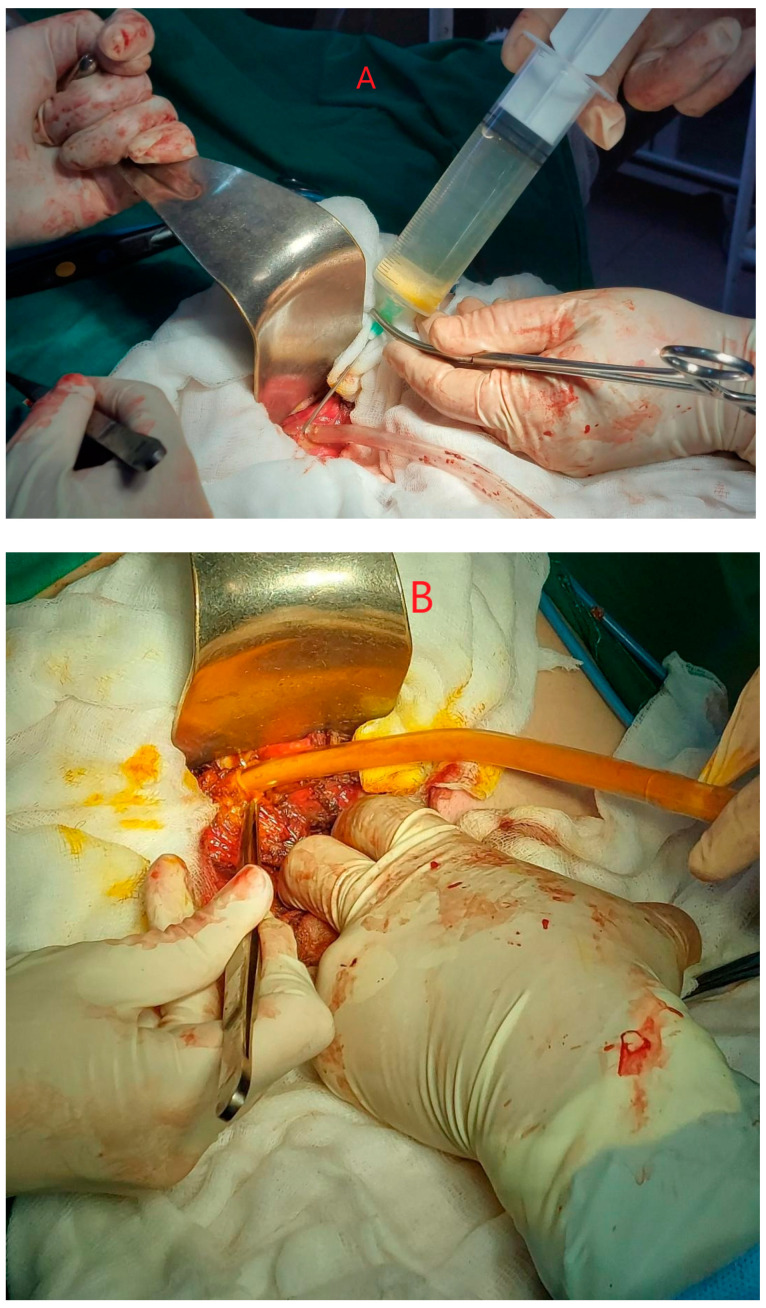
(**A**,**B**) Open approach for relapsing hydatid cyst inactivation and aspiration. Personal collection.

The strategy we chose was performed through a right subcostal or midline laparotomy. After identifying the hydatid cyst, the abdomen was isolated with soaked fields in a scolicidal agent. Puncture was performed, followed by evacuation of the cyst contents and instillation of the scolicidal agent (90% alcohol or hypertonic saline). A 10 min wait was allowed for the scolicidal agent to take full effect. The cyst contents were then evacuated. The intervention can proceed with either operculectomy, which involves removing the ex-tracapsular end of the cyst and draining the remaining cavity, or Lagrot pericystectomy, which involves more extensive removal of the cyst wall and cavity drainage. In the case of opting for ideal Napalkov cystectomy, the PAIR steps are exempted, the aim of this intervention being the ad integrum excision of the cyst (Figure 1).

#### 2.1.2. Laparoscopic Approach

An abdominal approach was performed in the standard version for the hepato-biliary laparoscopic approach, supplementing or placing the trocars differently if local examination imposed. The supra-mesocolic and perihepatic areas were isolated using fields soaked in scolicidal agent. The cyst was punctured with evacuation of the hydatid contents, a process occasionally impeded by the presence of the proligerous membrane or daughter vesicles, after which a scolicidal agent was instilled. The next step involved reaspiration of the remaining contents and evacuation of the cystic content using an endobag. The extraction of the cyst wall can be performed only in the visible extracapsular area through operculectomy or a laparoscopic pericystectomy may be attempted. Drainage of the remaining cavity is deemed mandatory.

#### 2.1.3. Laparoscopic Approach Using Special Instruments

**Figure 2:**
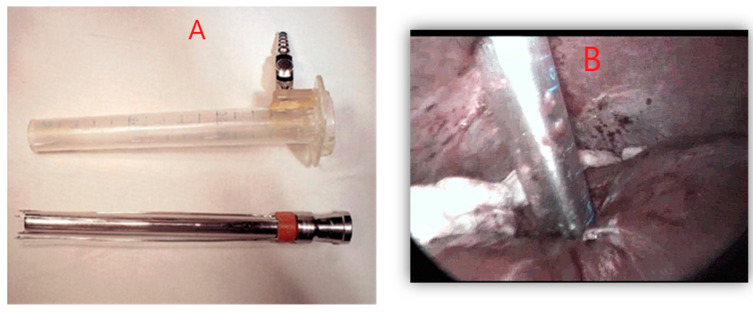
(**A**,**B**) Specialized devices with anchoring troacar on the cyst wall. Personal collection.

The procedure followed the principles of the standard laparoscopic approach and used the same type of instrumentation, with the addition of instruments specially de-signed for approaching the hydatid cyst. It is worth noting the coaxial trocar, through the presence of anchoring hooks, allows stable fixation to the cyst wall. With this trocar, all maneuvers corresponding to PAIR were performed in an isolated field with minimal risk of contamination. Additionally, the addition of a device for fragmenting the cystic contents reduced the risk of suction blockage (Figure 2).

The remaining steps of the intervention are comparable to the standard laparoscopic approach.

#### 2.1.4. ERCP

**Figure 3:**
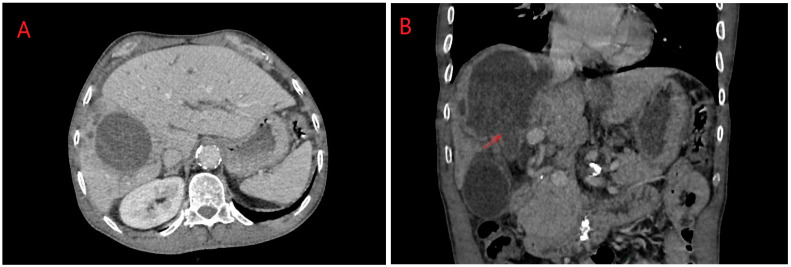
(**A**,**B**) Right lobe hydatid cyst rupture in right biliary duct: axial and coronal planes. Personal collection.

**Figure 4:**
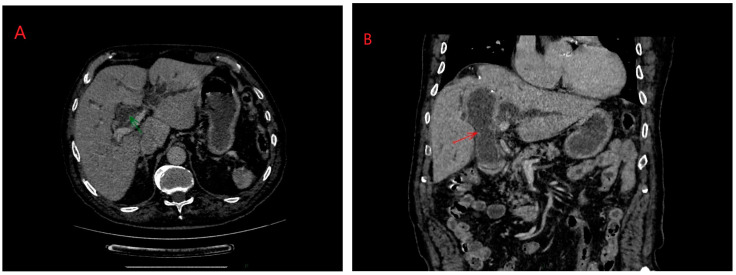
(**A**,**B**) Biliary rupture of liver hydatid cyst in main bile duct: axial and coronal planes. Personal collection.

**Figure 5:**
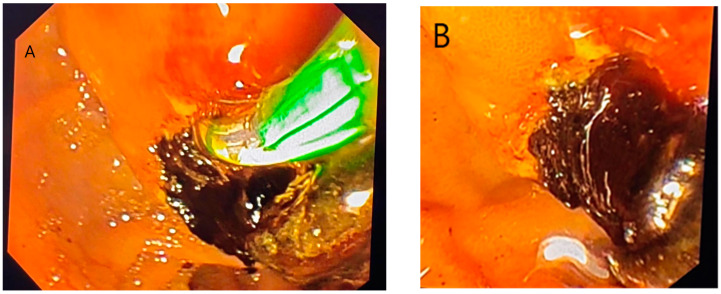
(**A**,**B**) ERCP steps for hydatid cyst rupture in bile duct: sphincterotomy and cyst layer removal. Personal collection.

ERCP (Endoscopic Retrograde Cholangiopancreatography) in hydatid disease is indicated in cases of cyst rupture into the main bile duct, a condition that can be diagnosed via CT/MRI imaging (Figure 3 and Figure 4) [14]. Obstruction caused by daughter vesicles, remnants of the proligera membrane, inflammation resulting from subsequent cholangitis can be resolved through this approach. After identifying and cannulating the duodenal papilla, radiological examination of the bile duct was performed, followed by sphincterotomy and extraction of the hydatid contents using a Dormia probe. Identifiable lesions in the bile ducts and inflammatory strictures can be stented, and with the maneuver, along with sphincterotomy, bile duct decompression can be achieved (Figure 5).

### 2.2. Statistical Analysis

Data for this study were collected from medical records and operative protocols. Statistical analyses was performed using Microsoft Excel 2016 (Microsoft Corp, Redmond, WA, USA), SPSS Statistics version 21.0, and DATA Tab. Numeric variables with a normal distribution were expressed as mean ± standard deviation and compared using Student’s *t*-test. Categorical variables were expressed as numbers (percentages) and compared using Fisher’s exact test. A *p*-value of less than 0.05 was considered statistically significant.

## 3. Results

During the study period, a total of 76 cases of abdominal hydatid cyst received surgical treatment in our hospital, out of which 18 were admitted with a complicated form of the disease.

Out of the total number of patients, 34.21% (*n* = 26) presented as emergencies, while the rest were electively admitted patients. Hydatid cyst complication cases accounted for 23.68% of all patients and 69.23% of those admitted as emergencies. The remaining cases, 30.76% (*n* = 8), were urgent admissions due to significant painful symptoms, without progressive complications caused by hydatid disease.

The analysis of cases revealed a higher frequency of this pathology among males, accounting for 57.89%, compared to females, who represented 42.11%. Hydatid disease was more commonly encountered in patients from rural areas (56.58%) compared to urban areas, where the frequency was 43.42%. The mean age was 51 ± 18.86 years, 52.52 ± 19.02 years for males and 48.91 ± 18.72 years for females.

A more detailed and breakdown presentation of the demographic data of the patients is presented in Table 1.

Of the patients from rural areas, 48.84% sought medical services in emergency, compared to 15.15% of patients from urban areas, with the latter primarily presenting by appointment at 84.85% (Chi^2^ = 9.41, df = 1, *p* = 0.002). The prevalent clinical sign was represented by abdominal pain (78.95%), followed by asthenia (9.21%), dyspeptic syndrome (6.59%), fever (3.95%), with only 1.32% of patients being diagnosed without any background symptomatology. A relationship was observed between the presence of algic symptoms being more frequent in rural patients (83.72%), compared to nonspecific symptoms (nausea, dyspeptic syndrome more frequent in urban patients (21.21%), without this ratio having statistical significance (Chi^2^ = 8.31, df = 7, *p* = 0.306).

Analyzing the number of hydatid cysts and their distribution at the hepatic or extrahepatic level, a higher frequency of single localizations (63.16%) was observed, most commonly in the right lobe of the liver, a similar situation being noted for multiple localizations.

Out of the total number of cases included in the study, 23.68% presented complications at the time of admission. In the group of cases with complications, the most frequent complication of the hydatid cyst was biliary rupture (33.33%), followed by cases of recurrence (22.22%), mass effect (16.67%), cyst infection (16.67%), and abdominal hydatidosis (11.11%). Cyst complications were more frequently encountered in cases with a single hepatic hydatid cyst (72.73%).

Cases with progressive complications of hydatid disease were admitted urgently in a larger number (57.14%), while non-urgent admissions represented 42.86% of the total cases with complications. Thus, there is a tendency for the initial manifestations of a complication of hydatid disease to have a subtle, urgent character. Statistical analysis confirms this observation (z statistic = 3.51, *p* < 0.001, OR = 0.35, 95% CI 0.19–0.63).

Additional details regarding the type of admission in relation to hydatid cyst complications are presented in Table 2.

Minimally invasive approaches (laparoscopy, laparoscopy with special instruments, and ERCP) represented the most common therapeutic approach, surpassing open surgery. Thus, open surgery accounted for 38.16% of all interventions, standard laparoscopy 34.21%, laparoscopy with dedicated instruments 26.32%, and ERCP approach 1.32%. In one case, therapeutic intervention was limited to ERCP due to associated patient comorbidities, but this method was frequently used as a step in the surgical treatment of hydatid cyst complications or postoperative complications.

Cases presenting with signs of acute abdomen complications, such as rupture of the hydatid cyst into the bile ducts, underwent open surgery as the primary operative intervention. This approach was preferred in 61.11% of all cases with complications of hepatic hydatidosis.

Table 3 synthesizes a distinct analysis of the complicated and uncomplicated cases.

Analyzing the relationship between the type of surgical approach and hydatid cyst complications, a connection was observed between cases with complications at the time of presentation and the type of approach, observing a higher frequency of the open surgical approach (r = 0.56, *p* = 0.02).

Open surgery was predominantly used in cases of hydatid cyst rupture into the biliary tract. Only one case was identified where this type of complication was treated laparoscopically, supplemented later with ERCP, and one case where management was limited to ERCP alone.

Abdominal hydatidosis was addressed through open surgery in two cases, with one case approached laparoscopically. Recurrences and cyst infections were predominantly managed through open surgery (Table 4).

Complicated hydatid cysts presented intraoperative complications such as hemorrhage, pneumothorax and diaphragmatic laceration. However, the analysis of cases without cystic complications did not detect statistically significant changes (r = 0.33, *p* = 0.153).

In cases with complications of the hydatid cyst, the most common approaches to the residual cyst cavity were maximal cystectomy (60.53%), Lagrot cystectomy (20.96%) and operculectomy (3.95%).

The average duration of surgical intervention in hydatid cyst complications was 164.85 min, higher compared to the average duration of interventions in the study (114 ± 71.71 min). Although a noticeable discrepancy was observed, no statistically significant changes were detected (ANOVA test-df = 1, F = 3.86, *p* = 0.063; Pearson correlation r = 0.04, *p* = 0.063).

Postoperative complications related to surgery occurred in 35.53% of patients, with the most common complication being biliary leakage (30.26%).

Complications due to the underlying disease and hospitalization occurred in 13.16% of patients, with the most common complication being Clostridioides difficile infection (7.89%). A relationship was observed between cases with surgery-related complications and complications of another nature (Chi^2^ = 11.88, df = 4, *p* = 0.018). Additionally, a statistically significant relationship was highlighted regarding the most frequent surgery-related postoperative complication (biliary fistula) and *Clostridioides difficile* infection (*p* = 0.029). The open surgery approach, especially through right subcostal laparotomy, was predisposed to the occurrence of late complications such as incisional hernia (*p* = 0.003), and a similar situation was observed in the comparison between laparotomy and recurrence (*p* = 0.003).

Analyzing the duration of hospitalization in comparison with the occurrence of postoperative complications revealed a statistically significant relationship between the two variables (Table 5).

In cases with complications of the hydatid cyst, a statistically significant association was found regarding the progression to multiple postoperative complications compared to cases without preoperative cystic complications (Chi^2^ = 17.18, df = 2, *p* = <0.001).

Complicated hydatid cysts are predisposed to a longer duration of surgical intervention, more frequent postoperative complications and a prolonged hospital stay.

The type of surgical approach in these cases also impacts the outcome, hospitalization and complications, with statistically significant results obtained in the comparative analysis between laparoscopy with dedicated instruments vs. open surgery (t = −4.08, *p* = 0.004) and between standard laparoscopy vs. open surgery (t = −3.22, *p* = 0.028).

## 4. Discussions

The current study aims to evaluate hepatic hydatid cysts in terms of the complications they can cause. The high frequency at which this parasitic disease leads to evolving complications (40%) underscores the importance of accurate and effective diagnosis and treatment management.

The evolution is usually asymptomatic or with nonspecific symptoms, but it can begin acutely in the form of complications such as anaphylaxis, acute abdomen, acute pancreatitis, jaundice, septic shock, ischemic or hemorrhagic vascular disorders, or rupture into the abdominal cavity or into the bile ducts.

Analyzing specialized literature by accessing international databases to gain a more comprehensive understanding of the complications of this pathology, we obtained 23 results, which have been synthesized in Table 6.

The literature analysis highlights the increased frequency of hydatid cyst complications such as rupture into the bile ducts, gallbladder, or compression of vascular structures. In fact, this type of complication is encountered in 15 out of the 23 reports included in the analysis. Other complications encountered include intraperitoneal rupture, mass effect and infection. The treatment of bile duct ruptures was predominantly represented by open surgery, with pericystectomy, bile duct exploration and drainage with a T-tube Kehr. The ERCP approach was the second therapeutic option chosen, but it was also used in some cases with T-tube drainage as an additional mechanism for biliary decompression.

Complications mainly consisted of bile fistulas and recurrence. The absence of complications in case reports is observed, while in retrospective analysis articles, the number of complications is significant. The explanation is provided by the importance of studying a significant sample and monitoring patients with this type of condition dynamically.

The most frequent complication detected in our study was the rupture of the hydatid content into the bile ducts (22.73%). This type of complication has an incidence ranging from 6–17% in patients with hepatic localized hydatid disease. Biliary ruptures are divided into frank rupture and occult rupture. In frank rupture, the hydatid material and daughter vesicles pass into the perforated bile duct and cause acute complications, more frequently jaundice, pancreatitis, or cholangitis. Occult ruptures, being more frequent, presuppose communication with low flow between the two elements; this communication most commonly results in biliary contamination of the cyst and its infection [42]. Frank rupture most commonly associates intense abdominal pain and jaundice. Rarely, it may associate acute pancreatitis, gallbladder hydrops, or acute cholecystitis. Prolonged release can lead to irreversible cholangitis with progression to biliary cirrhosis [33,43].

Imaging diagnosis is typically conducted through CT or MRI, with ERCP serving as an adjunct with both diagnostic and therapeutic value [44,45].

Treatment for this type of complication often involves an open approach and typically entails choledocotomy, exploration of the bile duct, extraction of hydatid contents, and external biliary drainage via a T-tube [34]. The procedure effectively clears the bile duct and relieves the patient of jaundice. However, it is burdened by prolonged biliary drainage, necessitating the use of ERCP to close the fistula. Prolonged hospitalization due to extended biliary drainage, a persistent fistula, or prolonged complications can lead to hospital-acquired infections, including multidrug-resistant organisms or disruption of the intestinal microbiota [46,47].

The less frequently used laparoscopic approach offers the benefit of shorter hospitalization duration and fewer complications. However, it requires preoperative or postoperative association with ERCP. The sole use of ERCP can yield good results, but it predisposes a patient to the risk of obstructive phenomena due to insufficient evacuation of the cystic cavity [14]. In the present study, the approach to this type of complication was carried out using each method described above. Most frequently, the use of a T-tube was associated with prolonged hospitalization and more frequent complications. Laparoscopy in combination with ERCP, as well as ERCP alone, yielded good results, but the small number of cases does not allow for definitive conclusions regarding the superiority of one method over the other.

The rupture of a hydatid cyst into the peritoneal cavity is a severe complication, leading initially to phenomena characteristic of an acute abdomen, septic state, or anaphylactic shock. In the long term, it can result in abdominal hydatidosis. While this condition happens to be secondary, yet in 2% of cases, it can be primary [48,49].

Surgical management of abdominal hydatidosis is almost exclusively performed through open surgery, with the laparoscopic approach being rarely used and dependent on the degree of dissemination [50].

Septic complications, compressions on biliary, vascular elements, or neighboring organs can be successfully addressed through laparoscopic or dedicated instrument approaches. The outcomes of minimally invasive approaches in these situations are superior to open surgery [51].

Our study highlights this aspect, demonstrating the utility of minimally invasive surgery in hydatid cyst surgery, both in uncomplicated forms and in the complications of the disease. Postoperative parietal defect complications, especially on right subcostal incisions leading to inefficient scar tissue formation, in patients who often have comorbidities or complications, represent a postoperative complication that should be avoided as much as possible [52,53].

Minor ruptures in the biliary tree can be effectively managed laparoscopically, with the absence of obstructive complications, mechanical jaundice or acute pancreatitis serving as an argument for the laparoscopic approach. In this situation, while evacuating the cyst with contact drainage, it is crucial to carefully select the scolicidal agent used (preferably hypertonic saline). This approach may be sufficient and could potentially eliminate the need for decompressive ERCP [54].

When a patient’s condition allows, obstructive cases involving hydatid material in the biliary or pancreatic ducts may benefit from initial ERCP intervention, with surgery considered as a subsequent step or performed remotely from the acute moment. In situations where access to an ERCP service is unavailable, severe cholangitis or jaundice, acute abdomen, or ineffective ERCP, an open approach is indicated. This typically involves external drainage using a T-tube and occasionally creating an anastomosis [55,56,57,58].

The management of infected hydatid cysts typically involves exclusively minimally invasive approaches, focusing primarily on evacuation and drainage. These procedures are effectively and comfortably performed using a laparoscopic approach. In situations where the infection originates from communication with the bile ducts and involves a substantial communication pathway, considerations may arise for endoscopic or surgical methods of biliary decompression [59,60,61].

Complicated hydatid cysts represent a medical reality, with the frequency of occurrence in the course of the disease and major local or systemic complications being a reason for efficient diagnosis and treatment, tailored to each type of complication and personalized according to the case’s characteristics.

## 5. Conclusions

Hepatic hydatid disease can lead to complications during its progression, necessitating modifications in therapeutic management that can affect the incidence of postoperative complications, thus increasing morbidity and mortality rates. Surgical intervention remains the cornerstone of treatment, with the choice between open or laparoscopic techniques guided by the nature and severity of complications. Additionally, employing ERCP as a therapeutic intervention for managing post-surgical complications offers substantial clinical advantages.

## Figures and Tables

**Table 1:** General characteristics of the patients.

Variable		Uncomplicated Hydatid Cyst (N = 58)	Complicated Hydatid Cyst (*n* = 18)	*p-Value*
Demographic characteristics				
Gender	Male	33 (56.92%)	12 (66.67%)	*p* > 0.05
	Female	25 (43.10%)	6 (33.33%)	
Age, yrs.		51 ± 18.86	58.16 ± 15.41	*p* > 0.05
Urban areas		26 (47.37%)	6 (33.33%)	*p* > 0.05
Presentation in emergency		8 (13.8%)	18 (100%)	*p* < 0.001
Location of the hydatid cyst				
Right lobe		36 (62.06%)	12 (66.66%)	*p* = 0.786
Left lobe		13 (22.41%)	2 (11.11%)	*p* = 0.498
Bilateral		9 (15.51%)	4 (22.22%)	*p* = 0.722

**Table 2:** Type of admission in relation with hydatid cyst complication.

	Admission Type		
Complication Type	Emergency	Programmed	Total
	*n* (%)	*n* (%)	*n* (%)
Diaphragm penetration	1 (4.55%)	2 (9.09%)	3 (13.64%)
Relapse, diaphragm penetration	0	1 (4.55%)	1 (4.55%)
Infected	2 (9.09%)	1 (4.55%)	3 (13.64%)
Bile duct rupture	5 (22.73%)	0	5 (22.73%)
Abdominal echinococcosis	1 (4.55%)	2 (9.09%)	3 (13.64%)
Relapse	2 (9.09%)	1 (4.55%)	3 (13.64%)
Infected, bile duct rupture	1 (4.55%)	0	1 (4.55%)
Left portal vein compression	0	1 (4.55%)	1 (4.55%)
Relapse, infection	0	2 (9.09%)	2 (9.09%)
Total	12 (54.55%)	10 (45.45%)	22 (100%)

**Table 3:** Type of surgical approach and hydatid cyst complications.

Variable	Uncomplicated Hydatid Cyst (58)	Complicated Hydatid Cyst (18)	*p-Value*
Open surgery	18 (31.03%)	11 (61.11%)	*p* = 0.022
Laparoscopic surgery	17 (29.31%)	3 (16.67%)	*p* > 0.05
Laparoscopic with special devices	23 (39.25%)	3 (16.67%)	*p* > 0.05
ERCP	0	1 (5.55%)	*p* > 0.05
Postoperative complications	26 (37.9%)	4 (22.22%)	*p* > 0.05
Hospital stay, days	11.67 ± 6.31	18.06 ± 15.94	*p* = 0.023

**Table 4:** Relation between approach type and complication type.

	Approach Type				
Complication Type	Dedicated Laparoscopic Instruments	Laparoscopy	Laparotomy	ERCP	Total
	*n* (%)	*n* (%)	*n* (%)	*n* (%)	*n* (%)
Diaphragm penetration	2 (9.09%)	1 (4.55%)	0	0	3 (13.64%)
Relapse, diaphragm penetration	1 (4.55%)	0	0	0	1 (4.55%)
Infection	0	1 (4.55%)	2 (9.09%)	0	3 (13.64%)
Bile duct rupture	0	1 (4.55%)	3 (13.64%)	1 (4.55%)	5 (22.73%)
Abdominal hydatidosis	0	1 (4.55%)	2 (9.09%)	0	3 (13.64%)
Relapse	0	0	3 (13.64%)	0	3 (13.64%)
Infected, bile duct rupture	0	0	1 (4.55%)	0	1 (4.55%)
Left portal vein compression	0	0	1 (4.55%)	0	1 (4.55%)
Relapse, infection	0	0	2 (9.09%)	0	2 (9.09%)
TOTAL	3 (13.64%)	4 (18.18%)	14 (63.64%)	1 (4.55%)	22 (100%)

**Table 5:** Statistical analysis for postoperative complications and hospitalization.

Pearson Correlation Analysis	r = 0.59, *p* < 0.001
*t*-test	t = −12.14, df = 75, *p* < 0.001, Cohen’s d = 1.39
Mann–Whitney U-Test	Z = −11.09, asymptotic *p = <0.001*, exact *p = <0.001*, r = 0.9

**Table 6:** Studies presenting the management and complications of hepatic hydatid cysts.

Authors	Complication Type	Patient Number	Management	Complications
Koc C et al. [20]	Intraperitoneal rupture Biliary rupture	29	Laparotomy, pericystectomy Bile duct exploration and T-tube drainage procedure	24.1% reccurence
Ammar H et al. [21]	Portal vein compression and rupture	1	Right hepatectomy	None
Elmajdoubi H et al. [22]	Gall bladder rupture	1	Laparotomy, cholecistetomy, trancistic drainage, perisistectomy	None
Borahma M et al. [23]	Bile duct rupture	1	ERCP	None
Al-Asbahi H et al. [24]	Bile duct rupture	1	Laparoscopic drainage	None
Adhikari S et al. [25]	Intraperitoneal rupture	1	Laparotomy	None
Ben Ismail et al. [26]	Inferior vena cava compression	1	Laparotomy Pericystectomy	None
Zenati H et al. [27]	Intraperitoneal rupture	1	Laparotomy Pericystectomy	None
Nayar R et al. [28]	Intrabiliary rupture	1	Left hepatectomy	None
Aghajanzadeh M et al. [29]	Intrabiliary rupture	1	Laparotomy, pericystectomy Bile duct exploration and T-tube drainage procedure	-
Önder RO et al. [30]	Intrabiliary rupture	1	ERCP	None
Mejri A et al. [31]	Intraperitoneal rupture	15	Laparotomy, pericystectomy	None
Biswas SN et al. [32]	Intrabiliary rupture	3	ERCP	None
Alimoradi M et al. [33]	Intrabiliary rupture	1	Laparomy, bile duct exploration and T-tube drainage	None
Toumi O et al. [34]	Intrabilary rupture	55	Laparotomy, pericystectmy Drainage 51 cases ERCP 4 cases	Fistula, Pulmonary infections, death. (*n* = 28)
Lahfidi A et al. [35]	Gall bladder rupture	1	Laparotomy, cholecystectomy, biliary drainage	None
Hamza A et al. [36]	Gall bladder rupture	1	ERCP, laparoscopic cholecystectomy	None
Leeuw D et al. [37]	Intrabiliary rupture	1	ERCP	None
Asadzadeh-Aghdaei H et al. [38]	Intrabiliary rupture	1	ERCP	None
Ahire P et al. [39]	Intrabiliary rupture	1	Laparoscopic pericystectomy, ERCP	None
Ufuk F et al. [29]	Intrabiliary rupture	1	ERCP, laparoscopic pericystectomy	None
Chopra N et al. [40]	Intrabiliary rupture	16	ERCP, laparoscopic pericystectomy	Biliary fistula (*n* = 5)
El Nakeeb A et al. [41]	Intrabiliary rupture	26	Laparotomy, T-tube, ERCP	Biliary fistula

## Data Availability

The datasets generated and analyzed during the current study are not publicly available due to institutional restrictions but are available from the corresponding author upon reasonable request.
